# Bone Mineral Density in Adults With Congenital Adrenal Hyperplasia: A Systematic Review and Meta-Analysis

**DOI:** 10.3389/fendo.2020.00493

**Published:** 2020-07-31

**Authors:** Swetha Rangaswamaiah, Vinay Gangathimmaiah, Anna Nordenstrom, Henrik Falhammar

**Affiliations:** ^1^Department of Diabetes and Endocrinology, The Townsville University Hospital, Townsville, QLD, Australia; ^2^Department of Endocrinology, Royal Darwin Hospital, Darwin, NT, Australia; ^3^Department of Emergency Medicine, The Townsville University Hospital, Townsville, QLD, Australia; ^4^Department of Women's and Children's Health, Karolinska Institutet, Stockholm, Sweden; ^5^Department of Pediatric Endocrinology, Astrid Lindgren Children's Hospital, Karolinska University Hospital, Stockholm, Sweden; ^6^Department of Endocrinology, Metabolism and Diabetes, Karolinska University Hospital, Stockholm, Sweden; ^7^Department of Molecular Medicine and Surgery, Karolinska Institutet, Stockholm, Sweden; ^8^Wellbeing and Chronic Preventable Diseases Division, Menzies School of Health Research, Darwin, NT, Australia

**Keywords:** bone mineral density, 21-hydroxylase deficiency, osteopenia, osteoporosis, glucocorticoids

## Abstract

**Background:** Decreased bone mineral density (BMD) is a concern in patients with congenital adrenal hyperplasia (CAH) due to lifelong glucocorticoid replacement. Studies till date have yielded conflicting results. We wanted to systematically evaluate the available evidence regarding BMD in adult patients with CAH.

**Methods:** We searched Medline, Embase and Cochrane Central Register of Controlled Trials to identify eligible studies. Studies comparing BMD in CAH patients with age- and sex-matched controls were included. Age <16 years and absence of controls were exclusion criteria. Two authors independently reviewed abstracts, read full-text articles, extracted data, assessed risk of bias using Newcastle-Ottawa scale, and determined level of evidence using Grading of Recommendations Assessment, Development, and Evaluation methodology.

**Results:** Nine case-control studies with a total sample of 598 (cases *n* = 254, controls *n* = 344) met eligibility criteria. Median age was 31 years (IQR 23.9–37) and 65.7% were female. Total body BMD (Mean Difference [MD]-0.06; 95%CI −0.07, −0.04), lumbar spine BMD (MD −0.05; 95%CI −0.07, −0.03) and femoral neck BMD (MD −0.07; 95%CI −0.10, −0.05) was lower in cases compared to controls. Lumbar spine *T*-scores (MD −0.86; 95%CI −1.16, −0.56) and *Z*-scores (MD −0.66; 95%CI −0.99, −0.32) and femoral neck *T*-scores (MD −0.75 95%CI −0.95, −0.56) and *Z*-scores (MD −0.27 95%CI −0.58, 0.04) were lower in cases.

**Conclusion:** BMD in adult patients with CAH was lower compared to controls. Although insufficient data precludes a dose-response relationship between glucocorticoid dose and BMD, it would be prudent to avoid overtreatment with glucocorticoids.

## Introduction

Congenital adrenal hyperplasia (CAH) is a group of autosomal recessive disorders of adrenal steroid biosynthesis (1, 2). 21-hydroxylase deficiency (21OHD) due to *CYP21A2* mutation is the most common cause of CAH accounting for 95–99% of all cases (3–5), followed by 11β-hydroxylase deficiency, 17α-hydroxylase/17,20-lyase deficiency, 3β-hydroxysteroid dehydrogenase type 2 deficiency, P450 oxidoreductase deficiency, lipoid adrenal hyperplasia, and cholesterol side chain cleavage enzyme deficiency (4, 6–8). Clinically classic 21OHD is characterized by glucocorticoid deficiency and adrenal androgen excess with or without additional mineralocorticoid deficiency (2). Incidence of classic CAH varies between 1:14000 and 1:18000 live births of which 75% are salt-wasting (SW) type and rest are simple virilizing (SV) (2). Non-classic 21OHD is more common with a reported incidence between 1:200 and 1:1000 livebirths, but even more common in certain ethnicities (9, 10). The treatment goals are to prevent adrenal crisis and optimize growth, sexual maturation, and reproductive function which is accomplished by replacing glucocorticoid and mineralocorticoid in sufficient doses (1, 2). This will decrease the associated excessive adrenocorticotropic hormone (ACTH) secretion from the pituitary gland and prevents hyperandrogenism. The balance between replacing deficient hormones and preventing hyperandrogenism can be difficult to achieve without overtreatment and its attendant risk of growth retardation and other clinical metabolic manifestations of glucocorticoid excess such as obesity, insulin resistance, diabetes, and hypertension (3, 4, 11, 12).

Decreased bone mineral density (BMD) and osteoporosis have been an important concern in patients with CAH due to lifelong glucocorticoid replacement. Glucocorticoids are a well-known secondary cause of osteoporosis and increased fracture risk has been established by various epidemiological studies (13, 14). Glucocorticoids have direct and indirect effects on bone leading to initial increased resorption and later decease in bone formation which results in microarchitectural distortion and increased fracture risk (15–17). It has also been postulated that glucocorticoids can cause secondary hyperparathyroidism by reducing intestinal calcium absorption and raising renal calcium excretion.

Gonadal and adrenal androgens are stimulators of osteoblast proliferation and differentiation in both males and females (18). Dehydroepiandrosterone sulphate (DHEAS) and other adrenal androgens affect bone metabolism throughout life and particularly during adrenarche, with a main effect on cortical bone (19). Some studies suggest that children with classic CAH fail to have a physiological rise in DHEAS levels during childhood, effectively accounting for absence of a typical adrenarche (20). Low DHEAS as a result of blunted response in adrenals of CAH patients and due to the glucocorticoid effect can affect the growth and osteoblastic function in these patients and might be the cause of low BMD.

However, there have been conflicting results in the published literature regarding BMD in CAH patients. Some studies reported normal BMD (21–27) while other studies reported low BMD in all or some sites (3, 28–44) or even high BMD (45). Thus, the aim of this systematic review and meta-analysis was to review the available literature to assess if adult patients with CAH are at risk of decreased BMD compared to age- and sex-matched controls.

## Methods

### Eligibility Criteria

Included studies had to have an exposure group consisting of patients aged 16 years or older with CAH, a control group consisting of age- and sex-matched controls without CAH, with standard BMD measures as outcomes of primary interest. Only clinical trials (RCTs and CCTs) and case-control studies were considered adapt to be included in this meta-analysis, eliminating any other study design such as studies with no age- and sex-matched controls, animal studies, reviews, editorials, news articles, case reports, case series, opinion pieces, and conference abstracts.

### Search Strategy

PubMed/Medline, Embase and Cochrane Central Register of Controlled Trials (CENTRAL) were searched from inception to 31st July, 2019 using the following keywords: “Congenital Adrenal Hyperplasia,” “CAH” “21-Hydroxylase,” “11β-hydroxylase,” “3β-hydroxysteroid dehydrogenase,” “17α-hydroxylase,” “Osteoporosis,” “Osteopenia,” “Bone mineral density,” “Bone densitometry,” “BMD” and “Bone metabolism.” For details, please see Supplemental Table 1. No language restrictions were applied. The reference lists of included studies and non-included reviews were manually searched to identify additional studies.

### Study Screening and Data Extraction

Four authors undertook the systematic review (SR, VG, AN, and HF) with SR coordinating the review. Two authors (SR and VG) independently ran the searches, screened the titles and abstracts as well as reviewed full-text copies to identify eligible articles. SR and VG independently performed data extraction using a standardized data extraction form (Microsoft Excel, Microsoft Inc, 2016). Data was extracted regarding the following variables: first author, type of study, country, year of publication, number of cases and controls, age, gender, whether the diagnosis of CAH was genetically confirmed or not, phenotype of CAH, body site, modality, and results of BMD measurements including *T*- and *Z*-scores, type, and average daily dose of glucocorticoid used for treatment, levels of bone turnover markers, type, and number of fractures, body mass index, vitamin D, 17-hydroxyprogesterone, androstenedione, testosterone, dehydroepiandrosterone, and dehydroepiandrosterone sulphate (DHEAS). Bone turnover markers are the collagen breakdown products and other molecules that are produced by osteoclasts and osteoblasts during bone resorption and bone formation. Markers that are specific to bone formation include serum bone-specific alkaline phosphatase (BALP), osteocalcin, and N-terminal propeptide of type I procollagen (P1NP), whereas markers specific to bone resorption include urinary N-terminal telopeptide of type I collagen (NTX), pyridinoline cross-links and serum C-terminal telopeptide of type I collagen (CTX).

An attempt was made to contact authors for clarification and additional data, if indicated. If such data could not be obtained, a decision was made to discuss its potential impact on the results. Disagreements were resolved by discussion and consensus. If consensus could not be reached, the last author (HF) made the final decision. The PRISMA (Preferred Reporting Items for Systematic Reviews and Meta-analyses) guidelines were followed (46).

### Data Synthesis

RevMan 5.3 was used to conduct data analysis. Results were calculated as mean differences with 95% confidence intervals (95%CI) for continuous data. *I*^2^ statistic was used to assess statistical heterogeneity between studies and significant heterogeneity was assumed if *I*^2^ was >40%. Meta-analyses using fixed-effect modeling were performed when the outcome data were found to be sufficiently clinically homogeneous. In the presence of significant heterogeneity, data was synthesized and presented qualitatively.

### Assessment of Risk of Bias of Individual Studies

The risk of bias of individual studies was assessed independently by two authors (SR and VG) using a combination of the Newcastle-Ottawa Scale (NOS) (47), Agency for Healthcare Research and Quality (AHRQ) standards (48) and the reviewers' interpretation of study quality. NOS was chosen due to its validity, interrater reliability and ease of use in case-control studies. NOS allows assignment of scores under three domains and eight sub-domains while AHRQ standards allow conversion of NOS scores to a quality assessment of studies as good, fair or poor. We interpreted good quality studies to be at a low risk of bias, fair quality studies to be at moderate risk of bias and poor-quality studies to be at high risk of bias. Publication bias was assessed graphically using funnel plots.

### Grading of the Body of Evidence

The quality of evidence was assessed independently by two authors (SR ad VG) using Grading of Recommendation, Assessment Development and Evaluation (GRADE) methodology (49) where evidence is graded on a quality continuum of high, moderate, low, and very low. GRADE methodology involves assigning a baseline quality of evidence based on study design which is usually low for observational studies and high for randomized trials with no significant limitations. Thereafter, there are five reasons for possible downgrading based on risk of bias, inconsistency, imprecision, indirectness, and publication bias. Reasons for possible upgrading include large magnitude of effect, dose-response gradient or if all plausible confounding would reduce the demonstrated effect or increase the effect if no effect was observed.

## Results

### Description of Studies

**Figure 1:**
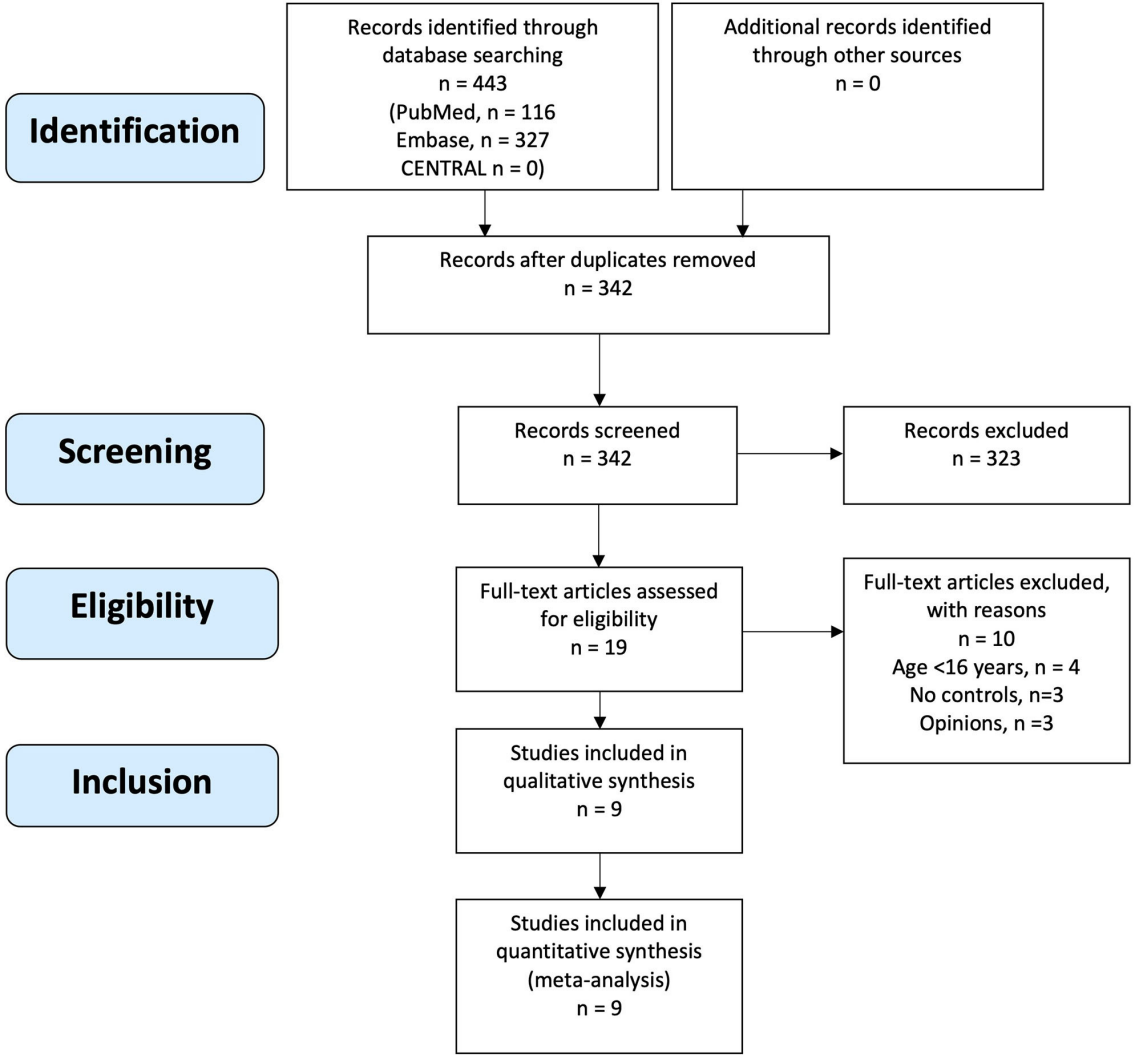
PRISMA flow diagram of study eligibility assessment and inclusion.

The initial search yielded 443 records of which 342 records were screened after removal of duplicates. Nineteen articles underwent full text review, of which nine articles met eligibility criteria and were included (Figure 1). Manual search of reference lists of included articles and non-included reviews did not yield any additional eligible articles.

### Included Studies

All nine included articles were case-control studies (23, 25, 33–37, 42, 43) with a total sample size of 598 (cases *n* = 254, controls *n* = 344). Median age of participants was 31 years (interquartile range 23.9–37) and 65.7% (393/598) were female. In all nine studies, cases were patients with CAH exposed to glucocorticoid use and controls were sex- and age-matched individuals without CAH. Outcomes were one or more measures of BMD. The characteristics of included studies are presented in Table 1.

**Table 1:** Characteristics of included studies of bone mineral density in adult patients with congenital adrenal hyperplasia and controls.

**Study first author and year of publication**	**Study location**	**Total sample size**	**Number CAH**	**Age CAH**	**Sex CAH**	**BMI CAH**	**Number controls**	**Age controls**	**Sex controls**	**BMI controls**	**Genetic diagnosis; CAH variant**	**DXA**	**BMD (g/cm^**2**^) cases vs controls**	***T*-score cases vs controls**	***Z*-score cases vs controls**
Ceccato 2016 (42)	Italy	76	38	31 ± 7 y	24M 14F	25.6 ± 5.9	38	31 ± 7	24M 14F	23 ± 3.4	Y 21OHD SW *n* = 21 SV *n* = 12 NC *n* = 5	Hologic	TB: (-) LS: 0.961+/-0.1 vs. 1.02+/−0.113 FN: (-)	TB: (-) LS: (-) FN: (-)	TB: (-) LS: −0.1+/−1.0 vs. −0.3+/−1.1 FN: (-)
Falhammar 2007 (36)	Sweden	122	61	24 y (18–29) 35 y (30–63)	F	22.4 (17.7-41.8) 24.4 (20.7-48.3)	61	24 y (18–29) 35 y (30–63)	F	21.9 (17.5–33.8) 23.7(19.4–39.9)	Y 21OHD SW *n* = 27 SV *n* = 28 NC *n* = 6	Mainly Lunar (Hologic *n* = 3)	TB: 1.128+/−0.016 vs. 1.186+/−0.015 LS: 1.130+/−0.025 vs. 1.237+/−0.025 FN: 0.941+/−0.025 vs. 1.039+/−0.028	TB: 0.03+/−0.20 vs. 0.77+/−0.19 LS: −0.57+/−0.20 vs. 0.32+/−0.21 FN: −0.27+/−0.19 vs. 0.45+/−0.23	TB: 0.0772+/−0.145 vs. 0.933+/−0.137 LS: −0.736+/−0.15 vs. 0.520+/−0.232 FN: −0.567+/−0.165 vs. 0.227+/−0.212
Falhammar 2013 (37)	Sweden	62	30	35.7 ± 11.4 y	M	26.4 ± 4.7	32	36.5 ± 11.9	M	24.5 ± 3.6	Y 21OHD SW *n* = 17 SV *n* = 11 NC *n* = 2	Lunar	TB: 1.17+/– 0.11 vs. 1.27+/– 0.10 LS: 1.16+/−0.20 vs. 1.23+/−0.16 FN: 0.96+/−0.13 vs. 1.05+/−0.14	TB: (-) LS: (-) FN: (-)	TB: (-) LS: (-) FN: (-)
Guo 1996 (23)	United Kingdom	22	11	38.3 ± 14.3 y	6F 5M	NR	11	38.3 ± 16.3 y	6F 5M	NR	Y 21OHD SW = 3, SV = NR, NC = NR; 11OHD *n* = 2	Lunar	TB: 1.14+/−0.11 vs. 1.176+/−0.19 LS: 1.196+/−0.13 vs. 1.054+/−0.19 FN: 0.95+/0.18 vs. 0.93+/−0.15	TB: (-) LS: (-) FN: (-)	TB: (-) LS: (-) FN: (-)
Hagenfeldt 2000 (33)	Sweden	26	13	23.9 ± 0.8 y	F	26.2 ± 1.7	13	22.3 ± 0.4	F	20.7 ± 0.3	Y 21OHD SW, *n* = 12 SV, *n* = 1	Lunar	TB: 1.12+/−0.02 vs. 1.13+/−0.02 LS: 1.14+/−0.04 vs. 1.13+/−0.03 FN: (-)	TB: (-) LS: (-) FN: (-)	TB: (-) LS: (-) FN: (-)
King 2006 (35)	USA	34	26	SW: 39 y (21–51) SV: 51 y, (32–71) NC: 9 y	F	24.5 (16.5–40.5)	9	49 (21–70)	F	19.5 (17.3–28.3)	Y 21OHD, SW, *n* = 11 SV, *n* = 15	Hologic	TB: 1.05+/−0.12 vs. 1.2+/−0.14 LS: 0.96+/−0.11 vs. 1.13+/−0.19 FN:(-)	TB: (-) LS: −0.61+/−1.26 vs. 1.14+/−1.59 FN: (-)	TB: (-) LS: −0.15+/−1.22 vs. 2.33+/−1.21 FN: (-)
Raizada 2016 (43)	India	27	15	27.5 ± 6.2y	F	28.9 ± 5.5	15	27.2 ± 5.2	F	27.8 ± 4.9	N SW, *n* = 2 SV, *n* = 13	Hologic	TB: (-) LS: 0.96+/−0.09 vs. 1.02+/−0.08 FN: 0.84+/−0.09 vs. 0.92+/−0.07	TB: (-) LS: −0.7 (−2.2 to 0.7) vs. −0.35 (−1.3 to 1.5) FN: −0.9 (−0.18 to 0.5) vs. 0.0 (−1 to 0.9)	TB: (-) LS: (-) FN: (-)
Sciann-amblo 2006 (34)	Italy	168	30	M: 22.8 ± 0.9 y F: 23.1± 0.8 y	15F 15M	24.3 (19.8–28.6)	138	22.8 ± 0.9	84 F 54 M	24.3 (19.3–45.2)	Y 210HD, SW, *n* = 24 SV, *n* = 6	Lunar	TB: 1.125+/−0.023 vs. 1.154+/−0.009 LS: 1.201+/−0.040 vs. 1.161+/−0.016 FN:	TB: (-) LS: (-) FN: (-)	TB: (-) LS: (-) FN: (-)
Stikkel-broeck 2003 (25)	Nether-lands	60	30	M: 21.7 ± 2.4 y, F: 20.6± 2.9 y	15F 15M	25 ± 3.6	30	21.9 ± 2.4	15F 15M	22.3 ± 1.9	Y 21OHD, SW, *n* = 24 SV, *n* = 3 NC, *n* = 3	Hologic	TB: 1.11+/−0.06 vs. 1.14+/−0.07 LS:1.01+/−0.08 vs. 1.05+/−0.09 FN: 0.95+/−0.15 vs. 0.89 (0.78–1.23)	TB: (-) LS: (-) FN: (-)	TB: (-) LS: (-) FN: (-)

*F, females; M, males; Y, yes; N, no; yrs, years; BMD, bone mineral density; TB, total body; LS, lumbar spine; FN, femoral neck; NR, Not reported; (-) reported for cases only precluding comparisons and conclusions; 21OHD, 21-hydroxylase deficiency; 11OHD, 11-hydroxylase deficiency; SW, salt wasting; SV, simple virilizing; NC, non-classic; NR, Not reported*.

### Methodological Quality

Three studies were found to be of poor quality (34, 42, 43), one (23) of fair quality and the rest were found to be of good quality (25, 33, 35–37) (Table 2). This was based on an initial score calculated using the NOS (47) which was then converted to a quality measure based on AHRQ standards (48). The reviewers then assigned high, moderate or low risk of bias to studies of good, fair, and poor quality, respectively.

**Table 2:** Risk of bias assessment of included studies based on NOS and AHRQ.

**Study**	**Selection**	**Comparability**	**Exposure**	**Quality**	**Risk of bias**
Ceccato 2016 (42)	4	2	1	Poor	High
Falhammar 2007 (36)	4	1	2	Good	Low
Falhammar 2013 (37)	4	1	2	Good	Low
Guo 1996 (23)	2	2	2	Fair	Moderate
Hagenfeldt 2000 (33)	4	1	2	Good	Low
King 2006 (35)	4	2	2	Good	Low
Raizada 2016 (43)	3	1	1	Poor	High
Sciannamblo 2006 (34)	4	1	1	Poor	High
Stikkelbroeck 2003 (25)	4	1	1	Good	Low

^36.^NOS-Newcastle Ottawa Scale. A study can be awarded a maximum of one star for each numbered item within the SELECTION and EXPOSURE categories while a maximum of two stars can be given for COMPARABILITY. The number items are as follows SELECTION 1. Is the case definition adequate? (a. yes with independent validation^*^ b. yes record linkage based on self-reports c. no description) 2. Representativeness of Cases (a. consecutive or obviously representative series^*^ b. potential for selection biases or not stated) 3. Selection of Controls (a. Community controls^*^ b. Hospital Controls c.no description) 4. Definition of controls (a. no history of disease^*^ b. no description of source) COMPARABILITY 5. Comparability of cases and controls on the basis of design or analysis (a. study controls for ___(select the most important factor)^*^ b. Study controls for any additional factor^*^) EXPOSURE 6. Ascertainment of Exposure (a. secure record like surgical records^*^ b. structured interview blinded to case/control status^*^ c. interview not blinded to case/control status d. written self-report or medical record only. e. no description) 7. Same method of ascertainment for cases and controls (a. yes^*^ b.no) 8. Non-response rate (a. same rate for both groups^*^ b. non-respondents described c. rate different and no designation) ^37^AHRQ-Agency for healthcare research and quality standards. Good quality: 3 or 4 stars in selection domain AND 1 or 2 stars in comparability domain AND 2 or 3 stars in outcome/exposure domain; Fair quality 2 stars in selection domain AND 1 or 2 stars in comparability domain AND 2 or 3 stars in outcome/exposure domain; Poor quality 0 or 1 star in selection domain OR 0 star in comparability domain OR 0 OR 1 star in outcome/exposure domain.

### Glucocorticoid Type and Mean Dose

The type of glucocorticoid used, average daily glucocorticoid dose and the results of included studies are shown in Table 3. Studies used between two to four different types of glucocorticoids with a proportion of patients in each study on more than one type of glucocorticoid for variable duration. Prednisolone was the most commonly used glucocorticoid. The average daily dose of glucocorticoid varied between 9.66 and 22 mg/m^2^/day hydrocortisone equivalents dose. For studies that reported individual patient glucocorticoid doses (23) or average daily dose without adjusting for body surface area (33), the average daily dose adjusted for body surface area was calculated using mean population adult body surface area for United Kingdom (50) and Sweden (51).

**Table 3:** Glucocorticoid type and hydrocortisone equivalent dose of patients with congenital adrenal hyperplasia in the included studies.

**First author and year**	**Type of glucocorticoids used for treatment and number of patients on each type**	**Average^**a**^ daily hydrocortisone equivalent dose (mg/m^**2**^/day)**	**Comments**
Ceccato 2016 (42)	Prednisolone, *n* = 1 Hydrocortisone, *n* = 3 Dexamethasone, *n* = 34	10+/−5	Cumulative glucocorticoid dose was not related to bone metabolism or BMD.
Falhammar 2007 (36)	Prednisolone, *n* = 30 Hydrocortisone, *n* = 17 Cortisone, *n* = 5 Dexamethasone, *n* = 7 Combination of two, *n* = 2	16.9+/−0.9	No correlations were found between BMD and the current glucocorticoid dose.
Falhammar 2013 (37)	Prednisolone, *n* = 18 Hydrocortisone/Cortisone, *n* = 8	17.4+/−5.2	No correlations were found between BMD and the current glucocorticoid dose. Patients on prednisolone had lower BMD than those on hydrocortisone/cortisone.
Guo 1996 (23)	Prednisolone, *n* = 2 Hydrocortisone, *n* = 3 Cortisone, *n* = 1 Dexamethasone, *n* = 5	17.4	The total glucocorticoid dose in the previous 2 years was not correlated with BMD
Hagenfeldt 2000 (33)	Prednisolone, *n* = 5 Cortisone, *n* = 1 Dexamethasone, *n* = 5 Triamcinolone, *n* = 1 Cortisone+Pred, *n* = 1	17.5	Negative correlation was found between BMD and the calculated index of accumulated post-menarcheal glucocorticoid dose
King 2006 (35)	Prednisone, *n* = 13 Cortisol, *n* = 10 Dexamethasone, *n* = 3	SW CAH: 22 (8–38) SV CAH: 17 (11–46) LS T-score < −1: 22 (13–28) LS T-score > −1: 15 (8–46)	There were not higher cortisol equivalents per body surface area among the osteopenic CAH patients compared to CAH patients with normal BMD
Raizada 2016 (43)	Prednisolone, *n* = 1 Dexamethasone, *n* = 14 (Pred+Dex, *n* = 6 Hydrocortisone+Dex, *n* = 5)	NR	Steroid doses not reported due to missing records
Sciannamblo 2006 (34)	Hydrocortisone (NR) Dexamethasone (NR)	F:15.3+/−1 M:17.1+/−1.1	No correlations were found between BMD and current glucocorticoid dose, nor the mean dose of the previous 7 yrs
Stikkelbroeck 2003 (25)	NR	F: 9.66+/−2.83 M: 13.16+/−2.66	No significant correlations were found between cumulative glucocorticoid doses in the last 0.5, 2, or 5 yr and bone parameters.

*NR, not reported; BMD, bone mineral density*.

a *average presented as mean+/- standard deviation or mean+/- standard error of mean or median(range)*.

### Meta-Analyses of BMD

**Figure 2:**
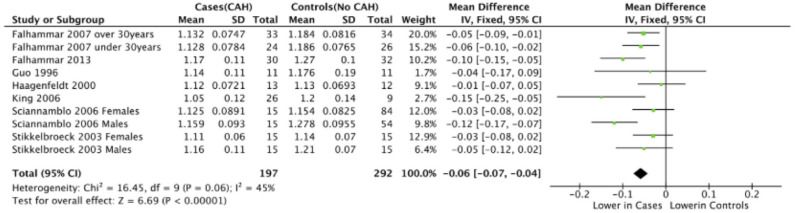
Meta-analysis of total body bone mineral density (g/cm^2^) in patients with congenital adrenal hyperplasia compared to matched controls.

**Figure 3:**
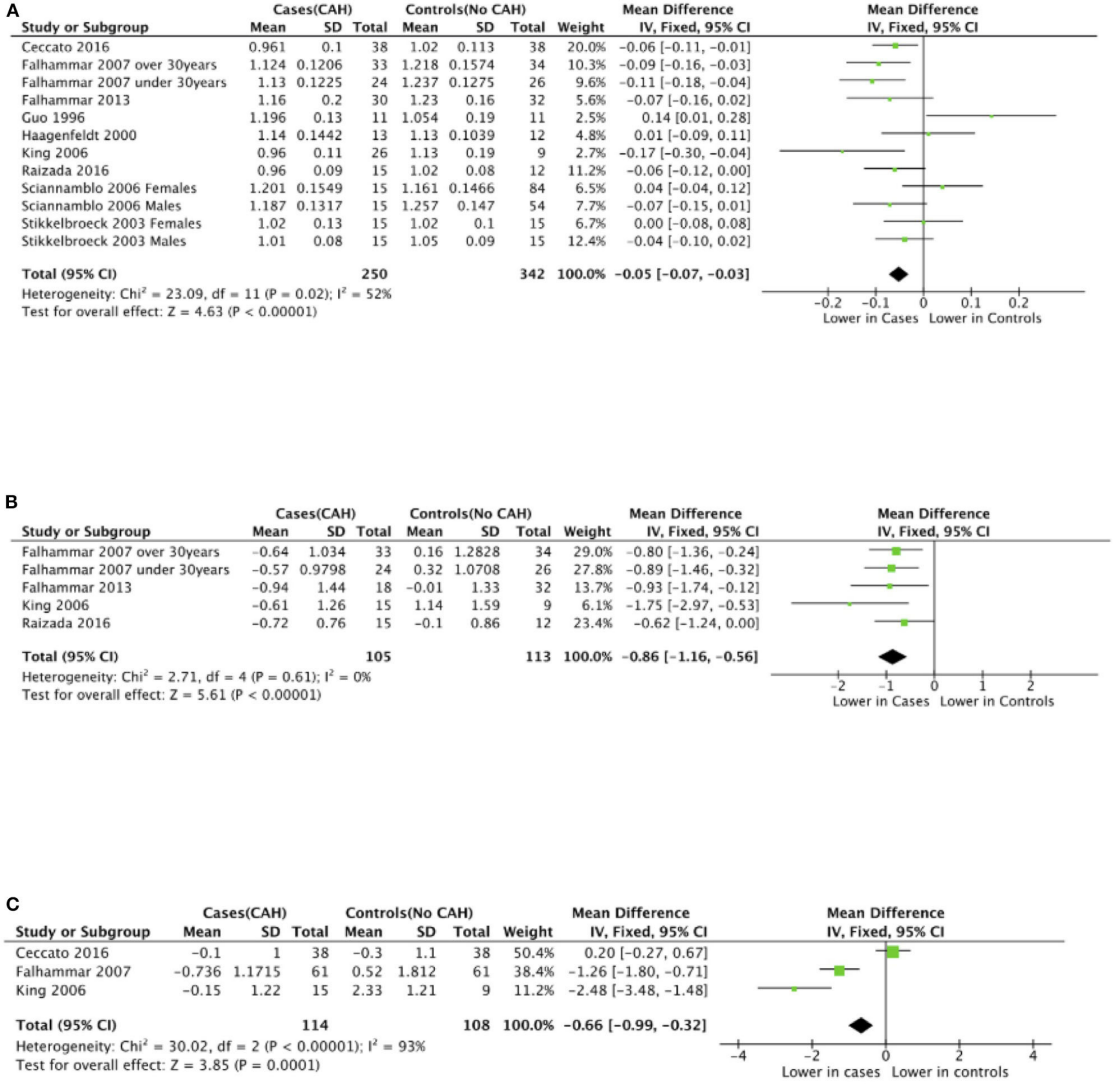
Meta-analysis of lumbar spine bone mineral density in patients with congenital adrenal hyperplasia compared to matched controls. **(A)** shows bone mineral density in g/cm^2^, **(B)** *T*-scores (*SD*), and **(C)** *Z*-scores (*SD*).

**Figure 4:**
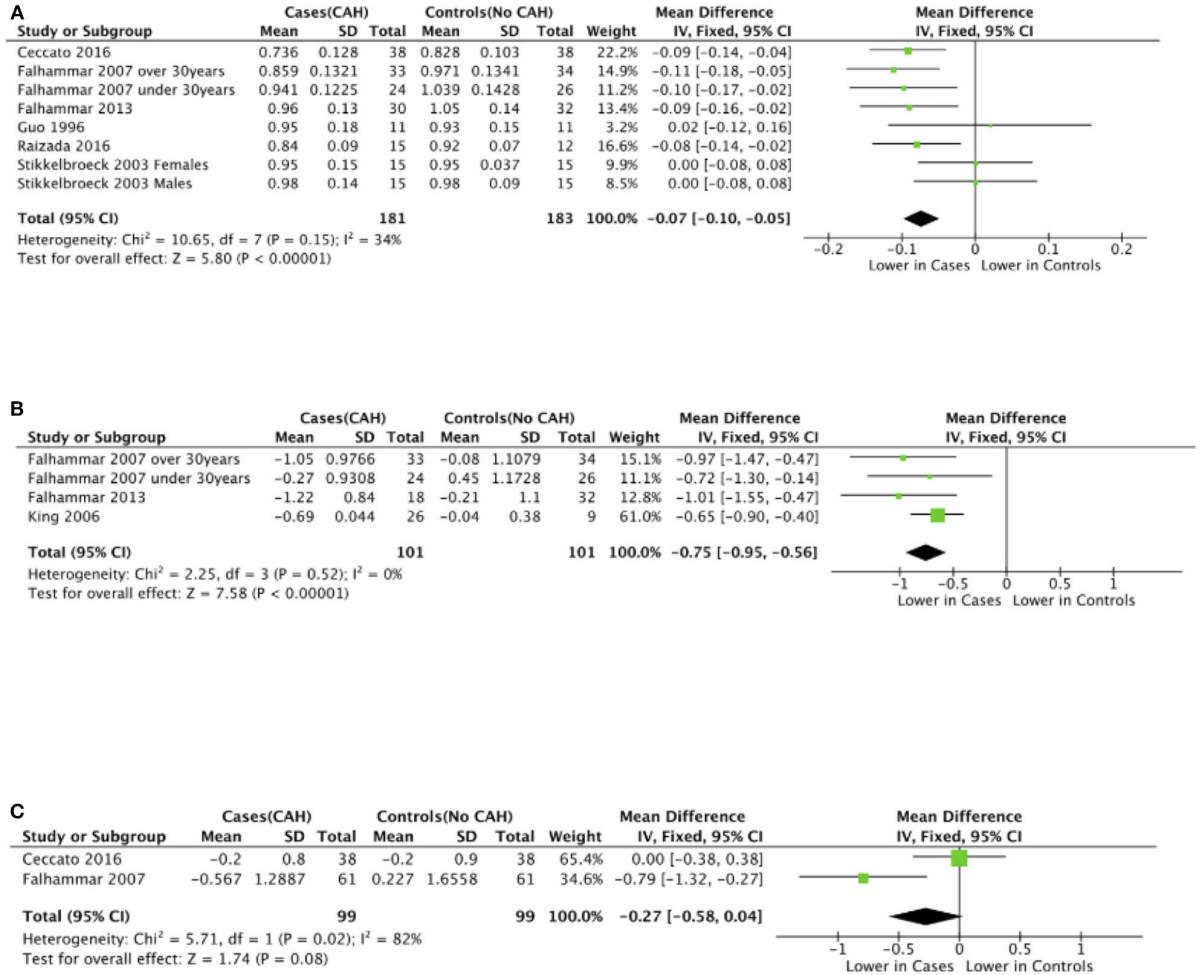
Meta-analysis of femoral neck bone mineral density in patients with congenital adrenal hyperplasia compared to matched controls. **(A)** shows bone mineral density in g/cm^2^, **(B)** *T*-scores (*SD*), and **(C)** *Z*-scores (*SD*).

Seven studies reported total body BMD (Figure 2) (23, 25, 33–37), nine studies reported lumbar spine BMD (Figure 3) (23, 25, 33–37, 42, 43), six studies reported femoral neck bone mineral density, (Figure 4) (23, 25, 36, 37, 42, 43) one study reported total femur BMD (42) and one study reported forearm BMD (43) Meta-analyses were performed for data variables where more than one study reported values for cases and controls. As some studies provided mean values for subgroups (males/females, <30 years/ >30 years) without an overall mean value, these subgroups were included as separate studies in the meta-analyses to minimize bias. Meta-analysis of the studies showed decreased BMD (g/cm^2^) at all commonly measured sites in CAH compared to controls with a mean difference for total body BMD of −0.06 g/cm^2^ (95%CI −0.07, −0.04), lumbar spine BMD of −0.05 g/cm^2^ (95%CI −0.07, −0.03), and femoral neck BMD of −0.07 g/cm^2^ (95%CI −0.10, −0.05). The *T*-scores and *Z*-scores (SD) at lumbar spine and femoral neck were also lower in cases compared to controls with a mean difference for lumbar spine *T*-score of −0.86 (95%CI −1.16, −0.56), lumbar spine *Z*-score of −0.66 (95% CI −0.99, −0.32), femoral neck *T*-score of −0.75 (95% CI −0.95, −0.56), femoral neck *Z*-score of −0.27 (95% CI −0.58, 0.04) (Figures 2–4, **Table 5**).

### Meta-Analysis of Bone Turnover Markers

**Figure 5:**
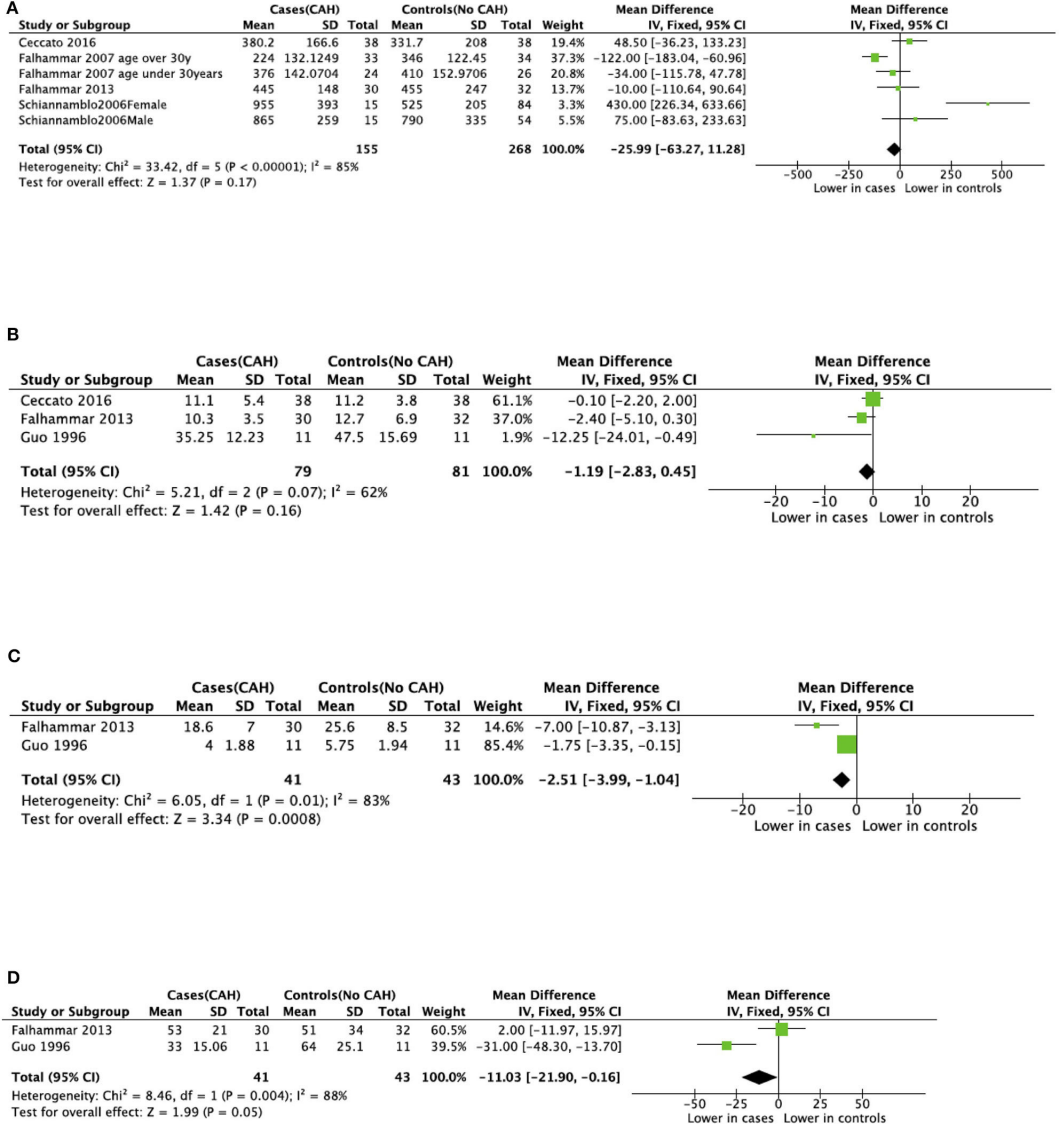
Meta-analysis of bone markers in patients with congenital adrenal hyperplasia (CAH) compared to age- and sex-matched controls. **(A)** Serum C-terminal telopeptide of type I collagen (CTX). **(B)** Serum Bone-specific alkaline phosphatase (BALP). **(C)** Serum Osteocalcin. **(D)** Urinary N-terminal telopeptide of type I collagen (NTX).

There was variable reporting and results of bone turnover markers in the included studies as shown in Table 4. CTX was reported in four studies (34, 36, 37, 42), BALP was reported in four studies (23, 36, 37, 42), osteocalcin and NTX were reported in two studies (23, 37). Meta-analyses of bone turnover markers showed that osteocalcin and NTX were lower in cases compared to controls whilst there was no significant difference in CTX and BALP between cases and controls (Figure 5). None of the studies reported P1NP or pyridinoline cross-links.

**Table 4:** Bone turnover markers in included studies of patients with congenital adrenal hyperplasia.

**First author and year of publication**	**CTX (ng/L)**		**BALP (units/L)**^****a****^		**Osteocalcin (mcg/L)**		**Urinary NTX/creatinine (nmol/mmol)**	
	**Cases**	**Controls**	**Cases**	**Controls**	**Cases**	**Controls**	**Cases**	**Controls**
Ceccato 2016 (42)	380.2+/−166.6	331.7+/−208	11.1+/−5.4	11.2+/−3.8	NR	NR	NR	NR
Falhammar 2007 (36)	<30y:376+/−29 >30y:224+/−23	<30Y:410+/−30 >30Y: 346+/−21	NR	NR	NR	NR	NR	NR
Falhammar 2013 (37)	445+/−148	455+/−247	10.3+/−3.5	12.7+/−6.9	18.6+/−7	25.6+/−8.5	53+/−21	51+/−34
Guo 1996 (23)	NR	NR	32 (19–58)	45 (25–75)	4 (1–7)	5.6 (2.8–9)	32 (10–58)	58 (30–110)
Raizada 2016 (43)	NR	NR	120	NR	NR	NR	NR	NR
Sciannamblo 2006 (34)	F: 900 (330–1,690) M: 850 (430–1,330)	F:460 (90–1,090) M:600 (310–1,650)	NR	NR	NR	NR	NR	NR

*NR, not reported*.

a *Units varied between studies: microg/l(Ceccato 2016), microkat/L(Falhammar 2007,2013), U/L(Guo 1996), IU/L Raizada 2016)*.

### Publication Bias

According to the Cochrane Handbook of Systematic Reviews for Interventions (version 6.0) “tests for funnel plot asymmetry should be used only when there are at least 10 studies included in the meta-analysis, because when there are fewer studies the power of the tests is low” and “none of the recommended tests for funnel plot asymmetry is implemented in RevMan.” As we used RevMan to perform all our meta-analyses and our systematic review includes nine studies, we are unable to comment on the possibility of publication bias.

### Sensitivity Analyses

The results of the meta-analyses remained unchanged when the analyses were limited to subgroups of female patients (Supplemental Figures 1–3).

### Grading of Evidence

The overall evidence was of low quality given the observational design of the included studies (Table 5). The evidence was downgraded for reduction in total body *T*- and *Z*-score, lumbar spine *T*- and *Z*-score as well as femoral neck *T*- and *Z*-score to very low quality due to imprecision of the effect size estimates resulting from wide confidence intervals. The evidence was not downgraded for reduction in total body BMD and lumbar spine BMD despite *I*^2^ > 40 due to clinical homogeneity of the included studies and the narrow confidence intervals around the effect size estimates. The evidence was not downgraded for risk of bias, indirectness, inconsistency, or publication bias. None of the studies met criteria for upgradation of quality of evidence for any of the outcomes.

**Table 5:** Summary of findings of included studies.

**Outcome measures**	**Results: absolute effects**		**Results: relative effects mean difference (95%CI)**	**Number of participants (studies)**	**Quality of evidence (GRADE)**	**Comments**
	**Cases**	**Controls**				
Total Body BMD	Mean total body BMD ranged from 1.05 to 1.17	Mean total body BMD ranged from 1.13 to 1.278	−0.06 (−0.07, −0.04)	489 (7)	Low	
Total Body *T*-score	Mean total body *T*-score ranged from 0.03 to 0.09	Mean total body *T*-score ranged from 0.73 to 0.77	−0.68 (−1.04, −0.33)	117 (1)	Very low^1^	Only one study reported Total Body T-scores in two age-based subgroups (<30 years and >30 years)
Total Body *Z*-score	Mean total body *Z*-score was 0.0772	Mean total body *Z*-score was 0.933	−0.86 (−1.25, −0.46)	50 (1)	Very low^2^	Only one study reported Total Body *Z*-scores in <30 years subgroup
Lumbar Spine BMD	Mean Lumbar Spine BMD ranged from 0.948 to 1.201	Mean Lumbar Spine BMD ranged from 0.991 to 1.257	−0.05 (−0.07, −0.03)	592 (9)	Low	
Lumbar Spine T-score	Mean Lumbar Spine T-score ranged from −0.94 to −0.57	Mean Lumbar Spine T-score ranged from −0.1 to 1.14	−0.86 (−1.16, −0.56)	218 (4)	Very low^3^	
Lumbar Spine *Z*-score	Mean Lumbar Spine *Z*-score ranged from −0.736 to −0.1	Mean Lumbar Spine *Z*-score ranged from −0.5 to 2.33	−0.66 (−0.99, −0.32)	222 (3)	Very low^4^	
Femoral Neck BMD	Mean Femoral Neck BMD ranged from 0.726 to 0.98	Mean Femoral Neck BMD ranged from 0.8 to 1.05	−0.07 (−0.10, −0.05)	364 (6)	Low	
Femoral Neck T-score	Mean Femoral Neck T-score ranged from −1.22 to −0.27	Mean Femoral Neck T-score ranged from −0.27 to 0.45	−0.75 (−0.95, −0.56)	202 (3)	Very low^5^	
Femoral Neck *Z*-score	Mean Femoral Neck Z-score ranged from −0.9 to −0.2	Mean Femoral Neck Z-score ranged from −0.3 to −0.2	−0.27 (−0.58, 0.04)	198 (2)	Very low^6^	

*Population, Adults(>18 years) with Congenital Adrenal Hyperplasia (CAH); Exposure, Therapeutic glucocorticoid use; Control, Age and Sex-matched adults without Congenital Adrenal Hyperplasia; Outcome, Risk of Osteopenia and/or Osteoporosis; BMD, Bone Mineral Density; CI, Confidence Intervals; GRADE, Grading of Recommendations Assessment Development and Evaluation. Footnotes about differences in subgroups age and gender based for each variable to be done 1,2,3,4,5,6: Level of Evidence downgraded from Low to Very Low for imprecision of effect size estimates secondary to small sample sizes and wide confidence intervals*.

## Discussion

This is the first systematic review and meta-analysis analyzing BMD in adult patients with CAH. The meta-analysis found consistently lower BMD at all commonly measured sites in individuals with CAH compared to age-and sex-matched controls.

The lower BMD in patients with CAH has been attributed to glucocorticoid overtreatment and the resulting catabolic effects of systemic glucocorticoids on bone in some studies (32, 33). The normal physiological cortisol production rate is estimated to be 9–11 mg/m^2^ (52), while the average glucocorticoid dose in the included studies was 9.66–22 mg/m^2^ hydrocortisone equivalents dose. Thus, most patients with CAH seemed to be on supraphysiological doses of glucocorticoids and this may be the main cause for low BMD. Hagenfeldt et al. found that the calculated index of accumulated post menarcheal glucocorticoid was the strongest determinant for all bone variables except BMD of spine and was calculated by multiplying the daily glucocorticoid dose expressed as cortisol equivalents with actual age minus age at menarche and dividing the result with the body surface area at the time of the study (33). King et al. suggested an association with oversuppression of adrenal steroidogenesis and decreased BMD in CAH patients (35). Similarly, Falhammar et al. found in both females and males with CAH subnormal levels of testosterone and DHEAS suggesting overtreatment with glucocorticoids which might have been a reason for low BMD (36, 37). However, most of the studies in our systematic review did not show any relationship between BMD and glucocorticoid dose. We could not perform a dose response analysis as some studies only had the current dose of glucocorticoids (36, 37) and those calculating a glucocorticoid dose over several years estimated the total dose from certain timepoint (23, 25, 33, 34, 42), (i.e., no study had the life-time dose exposure of glucocorticoids). Hence, we cannot draw any conclusions regarding dose response relationship.

Areal BMD as measured using DXA can be largely affected by size of bone leading to overestimation in larger bones and underestimation in smaller bones (53). CAH patients were 5–12 cm shorter than controls in studies included in our review. It has been shown that patients with CAH fail to achieve optimal adult height due to several factors (54). Excess adrenal androgens can result in accelerated linear growth and premature fusion of the epiphyses, ultimately compromising adult stature. Furthermore, treatment of CAH with glucocorticoids, even at replacement doses, especially around time of puberty has been associated with poor growth with resultant short stature in these patients (54). In our systematic review, two studies (36, 42) reported BMD corrected for height, two studies reported BMD corrected for BMI (35, 43) and the rest reported uncorrected BMD. In those correcting for height, Ceccato et al. found that only femoral neck BMD was lower in CAH patients compared to controls (42) while Falhammar et al. found that BMD remained lower in all studied sites in CAH patients compared to controls (36). King et al. and Raizada et al. found no association between BMD and BMI (35, 43). Given that only two of the included studies reported BMD corrected for height, it is not possible to draw any conclusions regarding the impact of height on differences in BMD observed between cases and controls.

Only one study reported BMD at forearm and there was no difference between cases and controls (43) However a recent study by El-Maouche et al. showed patients with classic CAH had lower BMD than patients with non-classic CAH, with the greatest difference at the forearm and DHEAS was the only independent significant predictor of BMD at the forearm, whole body and spine (19). We need further studies measuring cortical bone density to understand the etiology of these differences on cortical bone in CAH patients.

Sexual steroids increase osteoblast activity, decrease the formation, and activity of osteoclasts, stimulate longitudinal growth of long bones during puberty (55). The excessive estrogen action in pediatric CAH patients from peripheral conversion of androgens causes advanced maturation of the epiphyseal plate, resulting in increased bone age and acquisition of peak bone mass compared with healthy children (56). DHEAS correlates with bone turnover before peak bone mass (57), which may represent a direct effect on bone metabolism or the role of DHEAS as a substrate for conversion to other sex steroids. One of the aims of glucocorticoid therapy in CAH is to suppress the hyperandrogenism, however, often the androgen levels are lower than in controls (11, 37). Low levels of androgen as a result of glucocorticoid effect and failure of typical adrenarche in classical CAH patients have been shown to cause bone loss due to an imbalance between bone resorption and bone formation (58). Thus, androgens are important in both maintenance of bone mass and peak bone mass accrual.

Bone turnover markers were reported in 6 of the included studies with varied results. Osteocalcin and NTX were lower in cases than controls whereas CTX and BALP were not significantly different between cases and controls (23, 34, 36, 37, 42, 43). These conflicting findings could be due to variable age of study populations, assay differences, and different glucocorticoid preparations and doses.

No association was found between genotypes of CAH and BMD in three studies (33, 36, 37). The risk of fractures in CAH patients was reported by three of the included studies (36, 37, 43). No fractures were reported in any of the CAH cases by Raizada et al. (43). On the other hand, more fractures were found in CAH women than control (36), while no difference in fracture frequency could be demonstrated in males with CAH (37). However, fragility fractures usually occur in older age and very few patients with CAH above the age of 50 years were included in the studies in this review. Low BMD is strongly associated with the risk of fractures but it is well-recognized that different risk factors, such as age, history of a prior fragility fracture, steroid use and many others are independent contributors to the risk of fractures (59). Moreover, fractures can also occur due to sports activities, especially hazardous, which were more common in females but not in males with CAH (60, 61). One study reported that the fractures had often occurred during sport activities, however, the underlying trauma was not recorded systematically (37). There are also other studies reporting on fractures in CAH but then there was no control group making it difficult to assess the risk (19, 39, 44, 62). Thus, future studies examining fractures, how they occurred (fragility vs. traumatic fractures) in patients with CAH compared to controls are required.

Our systematic review and meta-analysis had several limitations. The relatively small total sample size of 598 makes it difficult to draw firm conclusions about the outcomes. As all included studies were retrospective observational studies, the possibility of results having been influenced by unknown confounders cannot be ruled out. Individual patient data was not assessed and numerical data from one study (23) was deduced from graphs possibly introducing measurement bias. However, the latter is unlikely to have influenced the direction and magnitude of results given the small sample size (*n* = 22) of the study. Despite the fact that only studies with CAH patients 16 years and older and controls were included, the study cohorts were still quite clinically heterogenous due to different genotypes of CAH, inclusion of both sexes, inclusion of different age groups and different DXA scans used to measure BMD. These factors could also have resulted in moderate degree of statistical heterogeneity as evidenced from the *I*^2^ statistic. Not all variables related to bone health were reported in each study and all the factors related to BMD could not be assessed. However, in all studies the controls were measured on the same DXA scan as their cases and all results point in the same direction reassuring that the conclusions are valid. Finally, inability to perform a glucocorticoid dose-response analysis and the low to very low quality of evidence for the reported outcomes in the included studies limits the strength of our conclusions.

### Future Directions

Bone health is an important issue. Well-designed, long-term prospective studies that assess all relevant measures of bone health in homogenous populations (age, sex, race, genotype) in relation to treatment are needed. Randomized studies are not possible instead structured long-term follow up are warranted, assessing glucocorticoid dose over time, and bone age during childhood years. Better endpoints than BMD would be fractures or markers of bone quality/strength but very few studies in CAH have investigated those, hence more studies are needed. The growth pattern (i.e., accelerated growth indicating insufficient dose and androgen excess or the opposite would also give important information). BMD in relation to glucocorticoid dose and hormonal control during puberty could give important insights to improve treatment strategies since puberty is thought to be the time in life for maximum bone mineral accrual. The timing of achieving peak bone mass in patients with CAH is lacking and timing of puberty is on average often earlier in this patient group. More data on peak bone mass in relation to puberty would be valuable. Bone markers would also be of importance to follow over time.

## Conclusion

BMD seemed to be consistently low in CAH patients compared to controls. Although we could not analyse the dose-response relationship between glucocorticoid dose and BMD, it would be prudent to avoid overtreatment. The current Endocrine Society Guidelines recommends BMD screening in any patient with CAH with a prolonged period of higher-than-average glucocorticoid dosing, or in patients who have had a non-traumatic fracture (2). The current systematic review and meta-analysis may support a more liberal BMD screening and monitoring in patients with CAH.

## Data Availability Statement

All datasets presented in this study are included in the article/Supplementary Material.

## Author Contributions

SR, VG, and HF: project conception and protocol preparation. SR and VG: idenfication of articles, data extraction, and data analysis. SR, VG, AN, and HF: manuscript preparation and review. All authors contributed to the article and approved the submitted version.

## Conflict of Interest

The authors declare that the research was conducted in the absence of any commercial or financial relationships that could be construed as a potential conflict of interest.
