# Supplementary material for: Bone Mineral Density in Adults With Congenital Adrenal Hyperplasia: A Systematic Review and Meta-Analysis
Source: Front Endocrinol (Lausanne). 2020 Jul 31;11:493. doi: 10.3389/fendo.2020.00493 (PMC7438951; doi:10.3389/fendo.2020.00493)

**Supplemental Table 1** Database search strategies

| **Database searched** | **Type of Search** | **Fields searched** | **Terms used** |
| --- | --- | --- | --- |
| PUBMED | Advanced | All fields  All fields  All fields | “Congenital Adrenal Hyperplasia” OR “CAH” OR “21-Hydroxylase” OR “11β-hydroxylase” OR “3β-hydroxysteroid dehydrogenase” OR “17α-hydroxylase”  AND  “Osteoporosis” OR “Osteopenia”  AND  “Bone mineral density” OR “Bone densitometry” OR “BMD” OR “Bone metabolism” |
| EMBASE | Advanced | Search | (“Congenital Adrenal Hyperplasia” OR “CAH” “21-Hydroxylase” OR “11β-hydroxylase” OR “3β-hydroxysteroid dehydrogenase” OR “17α-hydroxylase”) AND (“Osteoporosis” OR “Osteopenia”) AND (“Bone mineral density” OR Bone densitometry” OR “BMD” OR “Bone metabolism”) |
| CENTRAL | Advanced | All text  All text  All text | “Congenital Adrenal Hyperplasia” OR “CAH” OR “21Hydroxylase” OR “11βhydroxylase” OR “3βhydroxysteroid dehydrogenase” OR “17αhydroxylase”  AND  “Osteoporosis” OR “Osteopenia”  AND  “Bone mineral density” OR “Bone densitometry” OR “BMD” OR “Bone metabolism” |

CENTRAL, Cochrane Central Register of Controlled Trials.

**Sensitivity Analyses***

**Supplemental Figure 1**. Meta-analysis of total body bone mineral density in female patients with congenital adrenal hyperplasia compared to matched controls.


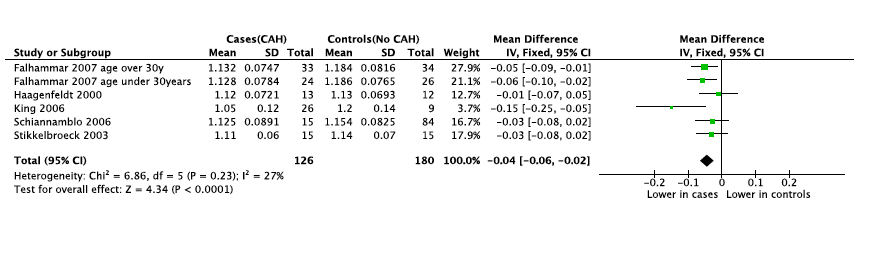


*6 female patients from Guo 1996 were not included in the sensitivity analyses as unique data from these patients was not available.

**Supplemental Figure 2**. Meta-analysis of lumbar spine bone mineral density in female patients with congenital adrenal hyperplasia compared to matched controls. Panel A shows bone mineral density (g/cm^2^), Panel B T-scores (SD) and Panel C Z-scores (SD).

A.


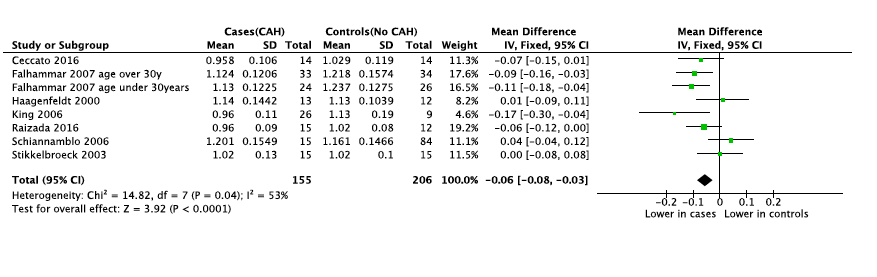


B.


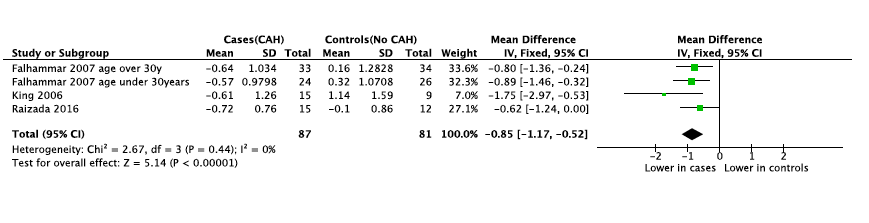


C.


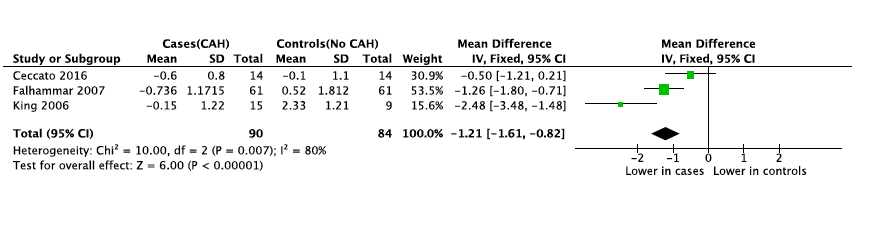


**Supplemental Figure 3**. Meta-analysis of femoral neck bone mineral density in female patients with congenital adrenal hyperplasia compared to matched controls. Panel A shows bone mineral density in g/cm2, Panel B T-scores (SD) and Panel C Z-scores (SD).

A.


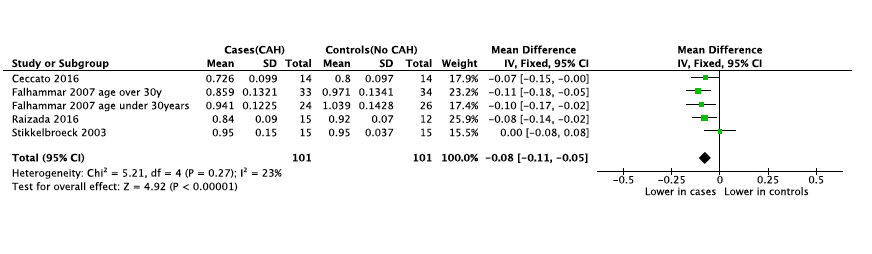


B.


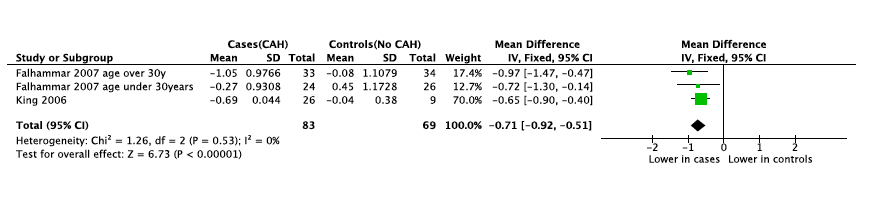


C.


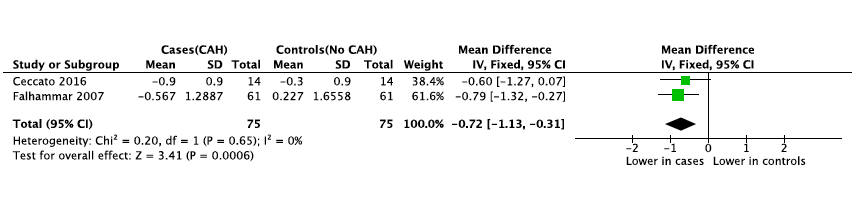

Supplement: Supplementary file 1 [file Table_1.docx]
